# Supplementary figures and images for: The Global Spread Pattern of Rat Lungworm Based on Mitochondrial Genetics
Source: Pathogens. 2023 May 31;12(6):788. doi: 10.3390/pathogens12060788 (PMC10300975; doi:10.3390/pathogens12060788)

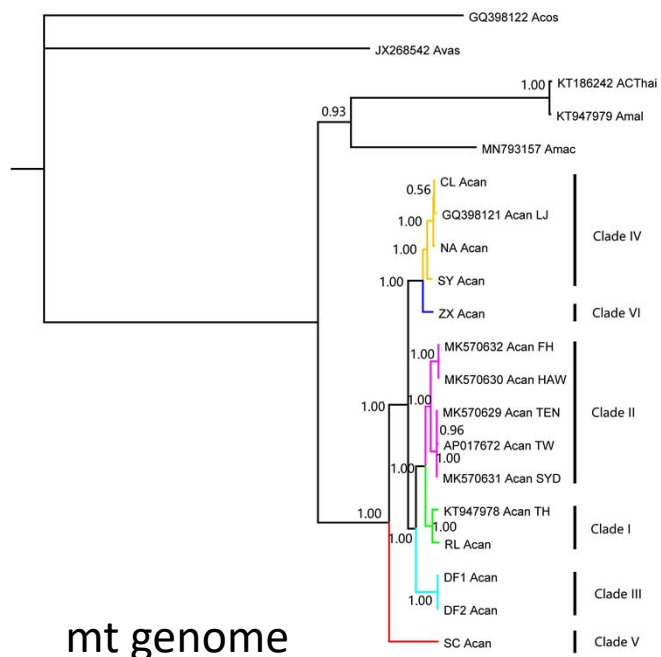

mt genome

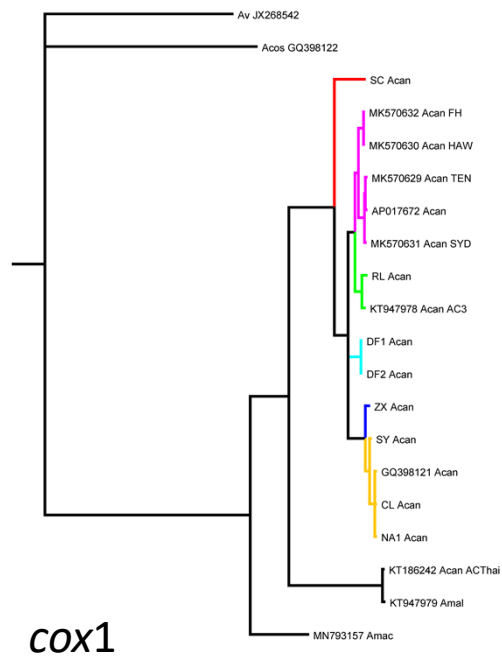

cox1

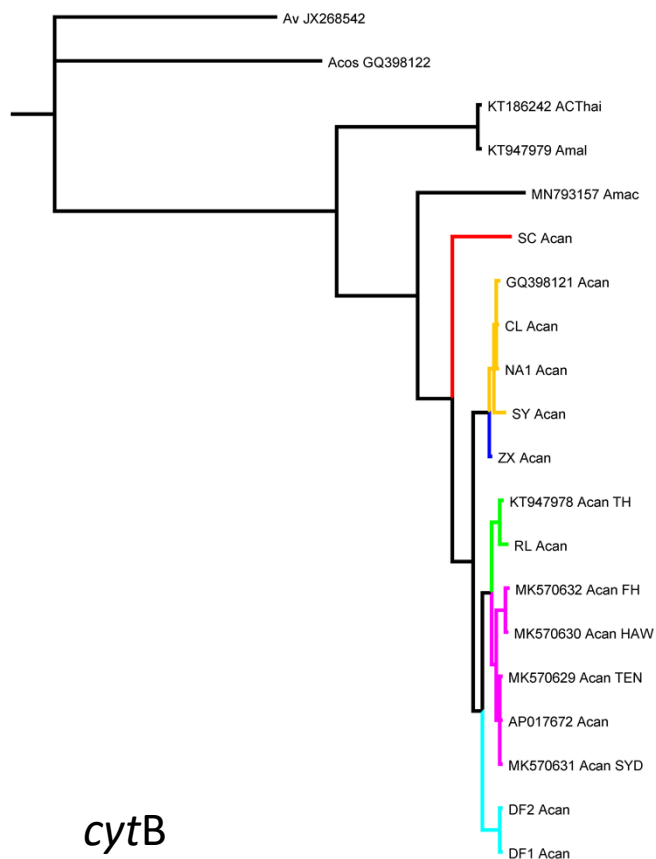

cytB

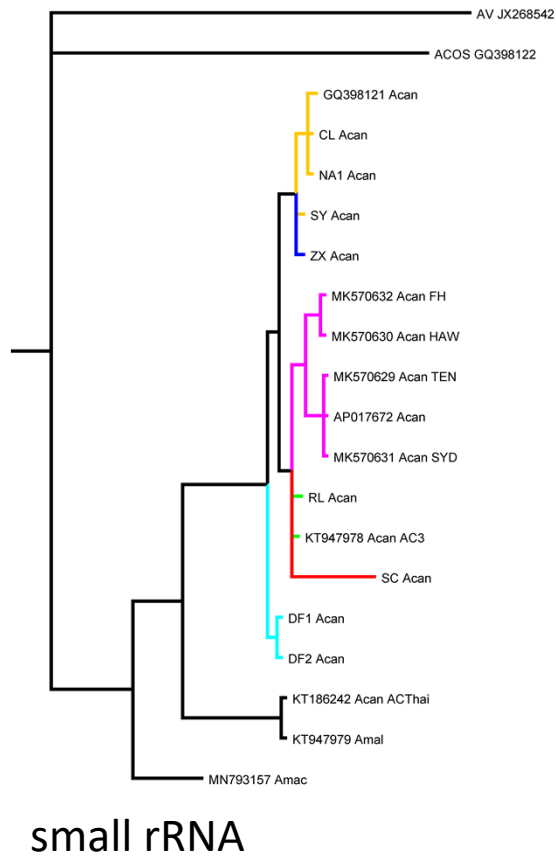

small rRNA

Supplement: Supplementary file 1 [file pathogens-12-00788-s001.zip › File S2. Comparison between trees based on mt genomes and individual genes.pdf]
